# Conservative management of pelvic abscess following sacrocolpopexy: a report of three cases and review of the literature

**DOI:** 10.1007/s00192-016-3189-z

**Published:** 2016-11-14

**Authors:** Soo Yun Kwon, Stacy Brown, John Hibbeln, Jeffrey Stephen Freed

**Affiliations:** 1 Department of OB/GYN, Rush University Medical Center, 1725 W. Harrison St. Suite 408, Chicago, IL USA; 2 Department of Radiology, Rush University Medical Center, Chicago, IL USA; 3 Department of Surgery, Icahn School of Medicine at Mount Sinai, New York, NY USA

**Keywords:** Abscess, Antibiotics, Drainage, Sacral colpopexy, Sacrocolpopexy, Mesh

## Abstract

**Introduction and hypothesis:**

After sacrocolpopexy, intra-abdominal pelvic abscesses are often managed with intravenous antibiotics, excision of the mesh involved, and debridement of compromised tissue.

**Methods and results:**

Three cases of successful management of pelvic abscesses after sacrocolpopexy using long-term antibiotics and percutaneous drainage of intra-abdominal abscesses without removing the mesh are presented.

**Conclusions:**

In selected patients who have undergone sacrocolpopexy, with careful counseling, conservative management of pelvic abscesses with percutaneous drainage and long-term antibiotic treatment without the surgical excision of the mesh may play a role.

## Introduction

Sacrocolpopexy has become the gold standard for the management of apical vaginal prolapse. In spite of prophylactic antibiotics and a careful surgical technique, pelvic abscesses remain disconcerting complications of pelvic reconstructive surgery. Reports indicate that mesh-related infection is an uncommon complication of pelvic organ prolapse repair: 0.7–8.0 % for vaginal mesh placement [1] and 0.3 % after laparoscopic mesh placement sacrocolpopexy [2]. When infection occurs after the placement of a mesh, such as in sacrocolpopexy, treatment usually involves resection of the mesh. Most published literature is based on case reports of treatment strategies and thus it is difficult to reach a conclusion regarding a standard treatment based on what is published. With the incorporation of type I, lightweight polypropylene mesh for sacrocolpopexy, however, it is possible that mesh resection may not be required in the face of a pelvic abscess. There are minimal data to necessarily support mesh resection for the treatment of pelvic abscess after vaginal mesh placement in all cases.

General surgeons have begun to investigate conservative management of abscesses associated with hernia mesh with significant success in selected patients [3]. These data have yet to be extrapolated to female pelvic reconstructive surgery. In three cases treated from one institution, performed by the same experienced surgeon (performing approximately 50 sacrocolpopexies per year) from 2012 to 2015, three patients with pelvic abscesses following sacrocolpopexy were successfully managed by CT-guided percutaneous abscess drainage followed by long-term broad-spectrum antibiotics. All our patients had received antibiotic prophylaxis at the time of the induction of anesthesia and the regimen was based on the American College of Obstetrics and Gynecology guidelines [4]. Each patient was administered cefazolin 2 g intravenously 1 hour before the skin incision. For surgery lasting more than 3 h, an additional intra-operative dose of cefazolin was given. Despite the fact that the patients underwent different approaches for their sacrocolpopexy, the same standard surgical techniques were used in all three patients including peritoneal covering of the mesh. The vaginal cuff was closed using V-Loc suture by Covidien, an absorbable barbed suture. The type I lightweight polypropylene mesh was attached to the vagina with Gore-Tex sutures. When a pelvic abscess did occur, each patient was extensively counseled and offered all treatment modalities (including surgery and mesh excision), and these three patients opted for management with drainage and intravenous antibiotics. The intravenous antibiotics were withheld until interventional radiology drainage was completed and the drain aspirate sent for culture. Institutional review board approval was not requested as this case series does not meet the Department of Health and Human Services (DHHS) definition of “research.”

## Case presentations

### Case 1

A 49-year old woman, G6P4, with stage 3 uterovaginal prolapse underwent total abdominal hysterectomy, abdominal sacrocolpopexy with lightweight type 1 polypropylene mesh and Burch urethropexy. She presented 1-week post-operatively with worsening pelvic pain and vaginal bleeding. Her temperature on presentation was 99.2 °F with a white blood cell count (WBC) of 11,200. Other vital signs were within normal limits. A CT scan of the abdomen/pelvis revealed an 11 × 9 cm pelvic abscess with extensive inflammation involving the sigmoid colon without evidence of bowel perforation. This finding prompted CT-guided drainage of the abscess, placement of a Jackson Pratt drain, and treatment with broad-spectrum antibiotics (piperacillin/tazobactam) for 9 days as per an infectious disease consultation based on organism sensitivity. The isolated organisms were polymicrobial. The patient was discharged home on oral levofloxacin and metronidazole for 16 days, as recommended by the infectious disease consultant. A follow-up CT scan of the abdomen/pelvis demonstrated complete resolution of the abscess before discontinuing the oral antibiotics. The patient’s 3-month follow-up examination revealed excellent vaginal support with no evidence of infection or mesh extrusion. The patient’s 6-month follow-up examination demonstrated excellent vaginal support and superficial, asymptomatic mesh extrusion managed by excision as an office procedure. The patient is presently sexually active at 2 years post-surgery and is without pain or further mesh extrusion.

### Case 2

A 56-year-old woman, G0, with stage 2 uterovaginal prolapse underwent robotic supracervical hysterectomy and sacrocolpopexy with lightweight type 1 polypropylene mesh. She presented 5 days post-operatively with fever of 101 °F and malaise. Her other vital signs were within normal limits and WBC was 13,000. A CT scan of the abdomen/pelvis demonstrated a 7.4 × 4.3 cm pelvic abscess and no evidence of bowel perforation. She underwent CT-guided drainage of the abscess, placement of a Jackson Pratt drain, and was started on broad-spectrum antibiotics (ampicillin/sulbactam and vancomycin). She was subsequently treated with piperacillin/tazobactam and vancomycin as per the infectious disease recommendation after identification and sensitivity studies of the infectious organism (*Bacteroides fragilis*). She was then discharged on hospital day #4 on oral levofloxacin and metronidazole for 21 days as per infectious disease recommendation. A CT scan of the abdomen/pelvis showed complete resolution of the abscess before the oral antibiotics were discontinued. The patient demonstrated excellent vaginal support at her 6-month follow-up visit. The patient reported being able to have intercourse without pain at the 2-year post-operative follow-up.

### Case 3

**Fig. 1:**
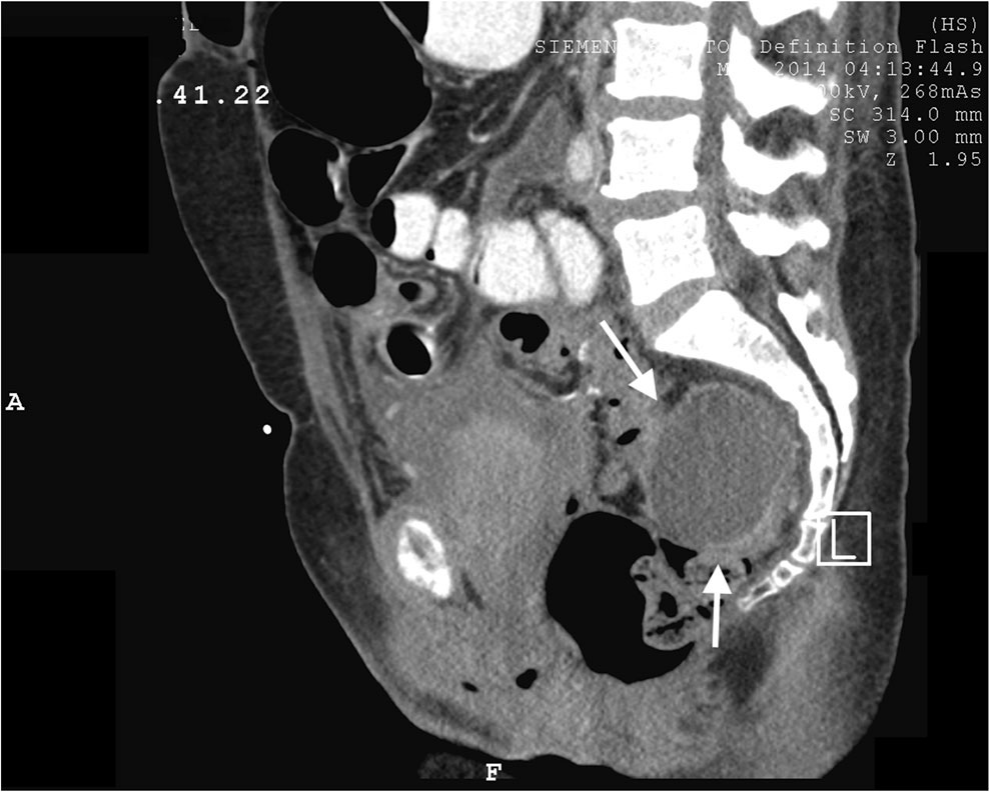
From patient #3. A sagittal midline image from the initial oral and intravenous contrast-enhanced CT. This demonstrates the rounded posterior pelvic fluid collection, 8.5 × 5.6 × 2.6 × 2.3 cm, with minimal marginal enhancement, consistent with an abscess (*arrows*)

**Fig. 2:**
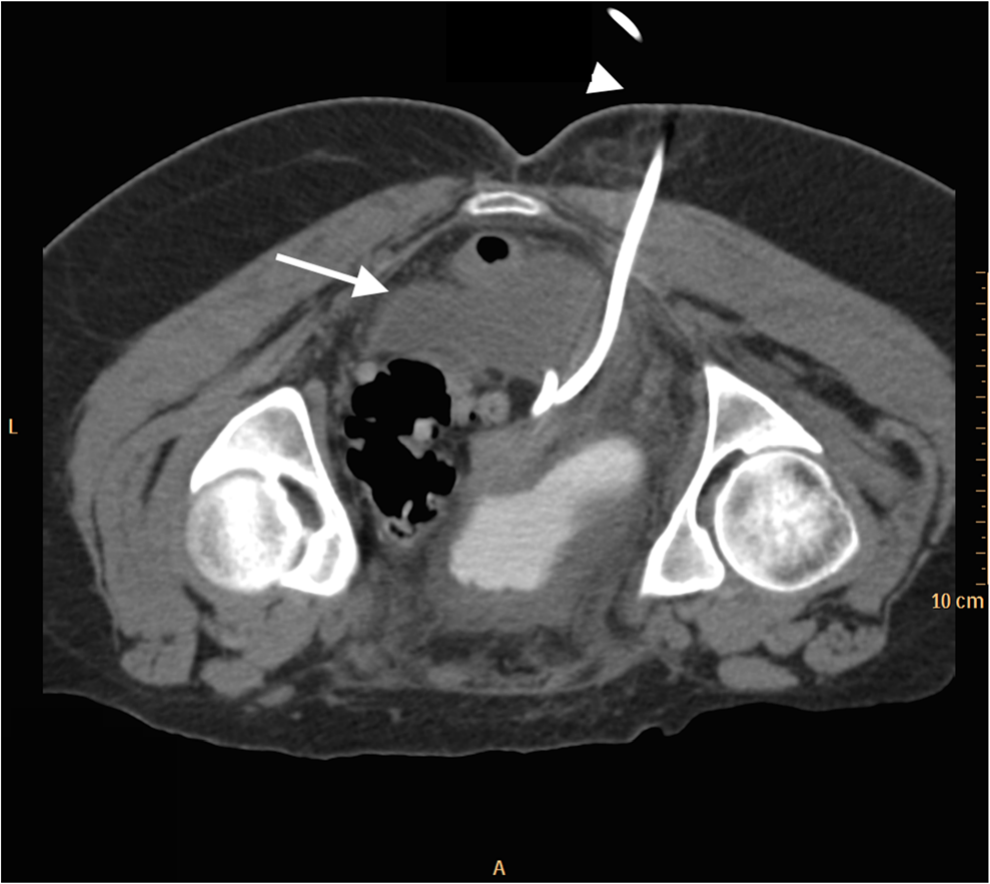
From patient #3. Transverse delayed post-contrast CT of the pelvis. The patient is in a prone position. There has been interval placement of a right-sided transgluteal drainage catheter (*arrowhead*) in to the posterior pelvic abscess (*arrow*), which was also seen in Fig. 1

**Fig. 3:**
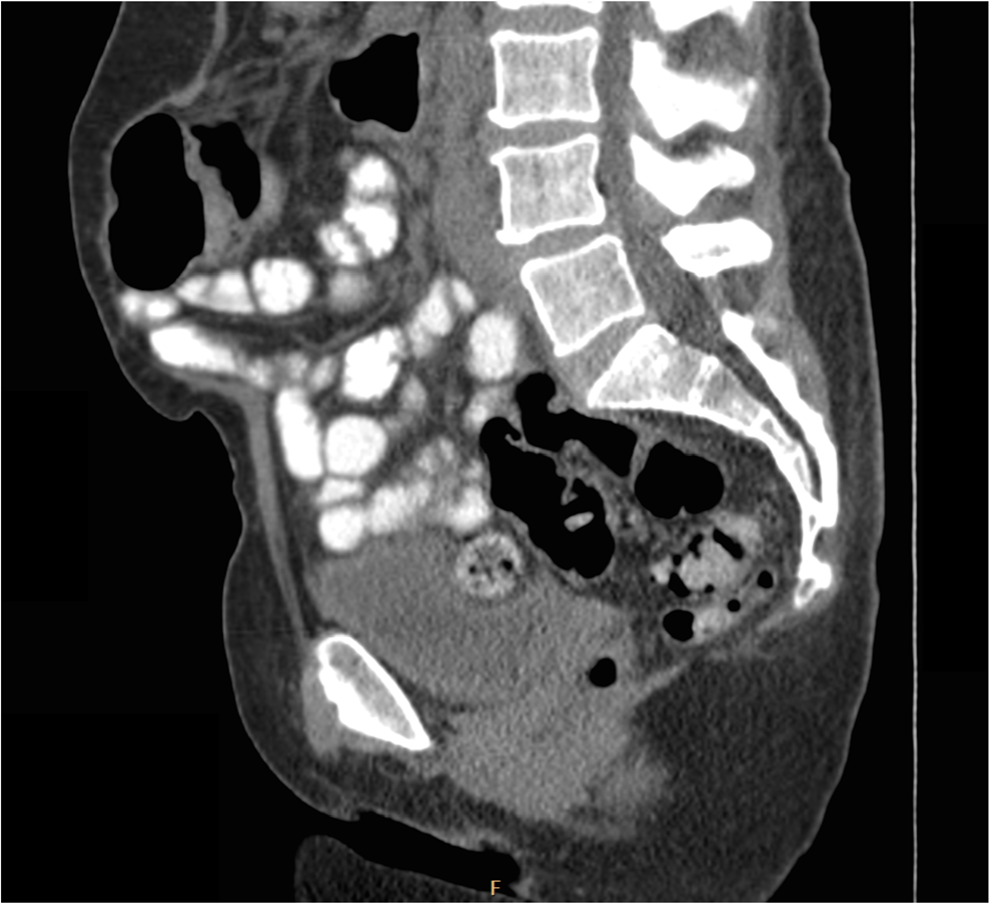
From patient #3. A sagittal midline image following catheter removal and long-term therapy with broad-spectrum antibiotics. There is interval resolution of the abscess previously seen in Fig. 1. The sigmoid is no longer displaced from the posterior pelvis

A 54-year-old woman, G5P4, with stage 3 vaginal prolapse underwent robotic sacrocolpopexy with lightweight type 1 polypropylene mesh, tension-free vaginal tape, and posterior repair. She presented 2 weeks post-operatively with abdominal pain and a WBC count of 16,000. She was afebrile with otherwise normal vital signs. A CT of the abdomen/pelvis demonstrated an 8.5 × 5.6 and 2.6 × 2.3 pelvic abscess, with no evidence of bowel injury (Fig. 1). The patient was offered surgical excision of the mesh, which she adamantly declined. She underwent CT-guided drainage of the abscesses and was discharged home on oral levofloxacin and metronidazole as per infectious disease recommendation based on the organism’s sensitivity (Fig. 2). The isolated pathogens were: *Citrobacter freundii*, *Streptococcus milleri* group, and *Bacteroides fragilis*. However, the patient did not complete the 14-day course of antibiotics. She subsequently presented to an outside hospital with abdominal pain and was diagnosed with a small bowel obstruction. She required an exploratory laparotomy and a small bowel resection for a compromised bowel, in addition to drainage of multiple abscesses. There was no evidence of iatrogenic bowel injury from the sacrocolpopexy reported by the operating surgeon. The patient was treated with intravenous vancomycin and meropenem as per the infectious disease recommendation. The antibiotic regimen was changed to ertapenem based on the sensitivity report. The isolated pathogens were *Escherichia cloacae*and *Escherichia aerogenes*. The patient was discharged home on intravenous ertapenem to continue for 2 additional weeks. She failed to continue taking the intravenous antibiotic, yet still declined surgical intervention. A follow-up CT scan demonstrated interval resolution of the pelvic abscess (Fig. 3); however, she was re-admitted 2 weeks after discharge with a recurrent pelvic abscess with no bowel perforation, which was again treated with percutaneous drainage and a 3-week course of inpatient intravenous antibiotics. The isolated pathogen was *Clostridium tertium*. A CT scan at that time demonstrated complete resolution of her abscesses. Of note, conservative treatment was chosen because the patient was a poor surgical candidate as per her general surgery consultation, in addition to her refusal to undergo additional surgery. At her 3, 6, and 12-month follow-up examinations, her vagina was noted to be well supported and had no evidence of mesh extrusion or infection. The patient did report dyspareunia at the 1-year follow-up.

## Discussion

We present three different cases of complicated post-operative pelvic abscesses following sacrocolpopexy, one patient with concomitant total abdominal hysterectomy, another with supra-cervical hysterectomy, and the third without hysterectomy (Table 1). All were successfully managed without resection of the sacrocolpopexy mesh. There was no evidence of mesh erosion into the visceral organs and only case #1 had superficial vaginal mesh extrusion.

**Table 1:** Comparison of patient characteristics

Patient	#1	#2	#3		
Body mass index	30.8	25.8	25.2		
Co-morbidities	Obesity, recent history of breast cancer	None	None		
Surgery	Total abdominal hysterectomy, abdominal sacrocolpopexy	Robotic subtotal hysterectomy and sacrocolpopexy	Robotic sacrocolpopexy		
Anti-incontinence procedure	Burch urethropexy	None	Mid-urethral retropubic tension-free vaginal tape sling		
Number of hospital admissions	1	1	1	2	3
Post-operative day number at presentation	8	5	14	60	84
Duration of hospital stay (days)	9	4	14	20	30
Cultured organisms	Polymicrobial	*Bacteroides fragilis*	*Citrobacter freundii*, *Streptococcus milleri* group, and *Bacteroides fragilis*	*Escherichia cloacae* and *Escherichia aerogenes*	*Clostridium tertium*
Inpatient intravenous antibiotic regimen	Vancomycin, piperacillin/tazobactam x 9 days	Vancomycin, piperacillin/tazobactam x 4 days	Ertapenem, Flagyl	Vancomycin, meropenem/ertapenem x 20 days	Ertapenem, Augmentin x 30 days
Outpatient antibiotic regimen	Levofloxacin, metronidazole x14 days	Levofloxacin, metronidazole x21 days	Levofloxacin, metronidazole x14 days (course not completed)	Metronidazole, ertapenem x 21 days (course not completed)	None

The likely etiology of the infections is unknown as the cultured pathogens were all different. Standard management of an abdominal or pelvic abscess after pelvic reconstructive surgery with mesh has previously been focused on total excision of the mesh, drainage of the abscess, and intravenous antibiotics [5]. Little consideration has been given to a possible role of non-operative management in selected patients. Unlike traditional management, our patients were treated using radiologically guided percutaneous drainage followed by continuous broad-spectrum antibiotics, without excision of the mesh. This resulted in complete resolution of the pelvic abscesses and prevention of further surgical morbidity, with no recurrence of vaginal prolapse.

When considering the non-operative treatment of pelvic abscesses after sacrocolpopexy, certain unknown factors should be kept in mind. Specific antibiotic regimens and lengths of treatment are uncertain, and the identification of appropriate patients for this management. In our patients, the choice and duration of the antibiotics was based on the recommendations by an infectious disease consultant. Conservative management may play a role in a subset of patients with infected mesh, to avoid further surgical morbidity. There is an established precedent in the general surgery literature for the conservative management of infected hernia meshes when the following clinical criteria apply:

1. Infection must be a localized abscess with no evidence of sepsis
2. No associated bowel injury must exist
3. No co-morbidity must be present that would indicate a poor outcome (e.g., immunosuppression) [6]

The patients in this case series demonstrated the criteria required to consider conservative treatment for a patient with a pelvic abscess after sacrocolpopexy with type 1 polypropylene mesh. Specifically, they had no bowel injury, they had a localized collection of purulent material amenable to percutaneous drainage, no evidence of sepsis, and no major co-morbidities. In our three patients, there was no evidence of bowel injury on their physical examination and evaluation by CT scan with oral/intravenous contrast medium. The third patient underwent a laparotomy with bowel resection for bowel obstruction, not related to any previous iatrogenic injury. Regarding the risk factors for the development of abscesses, the most likely cause of the abscess for patients #1and #2 is vaginal contamination from concurrent hysterectomy. Bacterial vaginosis has been associated with post-hysterectomy cuff cellulitis/infection due to the alteration of vaginal flora. Also, any laparotomy may be responsible for subsequent infection secondary to contaminating skin bacteria [7]. Patient #3 may have developed a hematoma that was secondarily infected.

Most research in the treatment of mesh infections has been done in general surgery patients who underwent mesh placement for ventral abdominal wall and inguinal hernias. The recommended management of mesh infections in this setting has been intravenous antibiotics, complete excision of the mesh, and drainage of all purulent collections [8]. In spite of the fact that it is rare to salvage an abdominal wall-infected mesh, there have been attempts to leave the mesh in situ and treat the infection by percutaneous drainage of any abscess along with intravenous antibiotics. Aguilar et al. [6] recently reported three patients with infected mesh secondary to localized abscesses salvaged after laparoscopic ventral hernia repair with the use of percutaneous drainage, antibiotic irrigation, and intravenous antibiotics. All patients have remained free of clinical signs of infection following the completion of this therapy. Aguilar et al. [6] concluded: “Infected mesh even with an abscess after laparoscopic ventral herniorrhaphy without systemic sepsis may be amenable to non-operative treatment.” Therefore, this conclusion led to the possibility that patients with a pelvic abscess after colpopexy may also be treated with percutaneous drainage followed by intravenous antibiotics, with the results possibly being retention of the prosthetic mesh in carefully selected patients.

In 1994, Amid et al. [9] was able to classify synthetic materials based on their properties, including pore size and fiber type [9]. This is of significance when considering a synthetic prosthesis for pelvic organ reconstruction. The advantageous properties of the mesh chosen should include: ease of use; facility of incorporating the host tissue with reduced risk for erosion, infection, and extrusion; and non-carcinogenicity. Monofilament, macroporous polypropylene mesh (referred to as type I or “lightweight”) is the synthetic material currently preferred for use in pelvic floor reconstruction and the large pore size (>75 μm) allows for in-growth of the mesh by macrophages, fibroblasts, and blood vessels. The resulting infiltration of the mesh that ultimately resembles and functions as native tissue promotes sound support for reconstruction and decreases the likelihood of infection [5]. Gore-Tex sutures are monofilament and microporous. There is no evidence in a review of the literature that these sutures have been reported to increase the likelihood of infection, unlike its counterpart, Gore-Tex mesh.

Although the incidence of pelvic abscesses after sacrocolpopexy is uncommon, the morbidity related to the necessity for mesh excision is significant. We have presented three cases of pelvic abscess after sacrocolpopexy performed via different surgical routes, using lightweight type 1 polypropylene mesh. All three infections resolved with percutaneous drainage of abscesses and intravenous and oral antibiotics (one patient requiring multiple treatments). It has been the standard of care to excise the mesh prosthesis if an abscess is present for the treatment of mesh infection. We have demonstrated that this may not be necessary in selected patients. A limitation of this paper is the small number of patients and follow-up of only 2 years. As reported by Collins et al. [10], infectious morbidities associated with mesh can occur many years after the surgery and a long-term follow-up will be necessary to conclude that conservative treatment is a viable option in a sub-group of patients. Also, larger studies are needed before this treatment can be considered a safe and effective modality. Questions to be answered in the future include the following. What are the criteria for patient selection? What is the appropriate number of attempts at percutaneous drainage? How do we initially choose an antibiotic and its length of treatment? When these questions are finally answered, percutaneous drainage of mesh-associated abscesses in pelvic reconstructive surgery may well become an accepted modality, resulting in markedly decreased patient morbidity.
